# Mammalian Solute Carrier (SLC)-like transporters of *Legionella pneumophila*

**DOI:** 10.1038/s41598-018-26782-x

**Published:** 2018-05-29

**Authors:** Ashley Best, Snake Jones, Yousef Abu Kwaik

**Affiliations:** 1 Department of Microbiology and Immunology, School of Medicine, University of Louisville, Louisville, KY United States; 2 Center for Predictive Medicine, University of Louisville, Louisville, KY United States

## Abstract

Acquisition of nutrients during intra-vacuolar growth of *L*. *pneumophila* within macrophages or amoebae is poorly understood. Since many genes of *L*. *pneumophila* are acquired by inter-kingdom horizontal gene transfer from eukaryotic hosts, we examined the presence of human solute carrier (SLC)-like transporters in the *L*. *pneumophila* genome using I-TASSER to assess structural alignments. We identified 11 SLC-like putative transporters in *L*. *pneumophila* that are structurally similar to SLCs, eight of which are amino acid transporters, and one is a tricarboxylate transporter. The two other transporters, LstA and LstB, are structurally similar to the human glucose transporter, SLC2a1/Glut1. Single mutants of *lstA* or *lstB* have decreased ability to import, while the *lstA/lstB* double mutant is severely defective for uptake of glucose. While *lstA* or *lstB* single mutants are not defective in intracellular proliferation within *Acanthamoeba polyphaga* and human monocyte-derived macrophages, the *lstA*/*lstB* double mutant is severely defective in both host cells. The two phenotypic defects of the *lstA*/*lstB* double mutant in uptake of glucose and intracellular replication are both restored upon complementation of either *lstA* or *lstB*. Our data show that the two glucose transporters, LstA and LstB, are redundant and are required for intracellular replication within human macrophages and amoebae.

## Introduction

Legionnaire’s disease, an atypical pneumonia, is a result of inhalation of the bacteria *Legionella pneumophila*^1–3^. Within the human host, *L*. *pneumophila* primarily reside and replicate within alveolar macrophages^4–6^. Infection of humans is considered to be “accidental”, as the natural hosts for *L*. *pneumophila* are protozoa in the aquatic environment^7,8^. Growth within either host occurs through manipulation of evolutionarily conserved pathways, to avoid fusion to the lysosomes and to remodel the vacuole to become ER-derived, which is designated as the *Legionella*-containing vacuole (LCV)^9–14^. The Dot/Icm type IVb translocation system, which translocates >320 effector proteins into the host cytosol, is required for biogenesis of the LCV^15^ and for successful intracellular replication in macrophages and amoebae^13,16–19^, and subsequent killing of the host cell^20^. A plethora of host cell processes are modulated by the translocation of redundant effector proteins^21^ that allow *L*. *pneumophila* to evade innate immunity and acquire nutrients^22–25^.

*L*. *pneumophila* relies on host amino acids (such as, serine, cysteine, and alanine) to feed into tricarboxylic acid (TCA) cycle as the main source of carbon and energy^26–28^. The bacteria are in such high demand for amino acids that endogenous amounts within the host are below the threshold needed to support robust intracellular replication^5,22,29^. To raise host cellular levels of amino acids, *L*. *pneumophila* translocates the AnkB effector, which is post-translationally modified by the host cell^30–32^, and hijacks the host ubiquitination-proteasome machinery to degrade proteins^22,33–35^, but the host cell also undergo metabolic reprograming in response to infection^36^.

Early studies pointed to a preference for amino acids as an energy source and *in vitro* studies identified auxotrophies for seven amino acids in *L*. *pneumophila*: threonine, arginine, isoleucine, methionine, leucine, cysteine, and valine^26,37^. Many of these auxotrophies are shared with the amoeba host which may allow the bacterium to synchronize growth with that of the host, avoiding deleterious growth during times of environmental stress^38–40^. *Legionella* can enter into a viable but non-culturable (VNBC) state when encountering nutritional stress, which has only been shown to be recovered by co-culturing with amoebae^41^.

Nutritional virulence studies on *L*. *pneumophila*^29,42,43^ have focused on the generation and utilization of amino acids^22,27,42,44,45^. Only recently has glucose metabolism been studied for its role during intracellular replication^46,47^. However, supplementation of glucose *in vitro* does not enhance growth of *L*. *pneumophila*^47^. Glycolysis plays a minimal role in glucose catabolism, but is predominately metabolized through the ED pathway while the pentose phosphate pathway (PPP) functions only to generate mannose and histidine^46–48^. A gene cluster encoding enzymes for glucose catabolism, through the Entner-Doudoroff (ED) pathway of *L*. *pneumophila* has been shown to be required for growth in the A549 epithelial cell line, A/J mouse macrophages, and *Acanthamoeba culbertsoni*, indicating the importance of the ED pathway in intracellular replication of *L*. *pneumophila*^47^. Initial studies focused on poly-3-hydroxybuyterate (PHB), a 4-carbon storage molecule that is generated by metabolizing glucose, through the ED pathway into pyruvate, which gets converted into acetyl-CoA, then PHB^49^. PHB is synthesized in late stages of growth and catabolized during stationary growth into acetyl-CoA to feed into the TCA cycle^49,50^. The primary usage of glucose by *L*. *pneumophila* is considered to be conversion into PHB^46,50^.

How nutrients are imported by *L*. *pneumophila* is not well understood^51,52^. To date, only one amino acid transporter, PhtA, of *L*. *pneumophila* has been shown to import threonine and is required for intracellular replication in macrophages^53^. Given that numerous genes in *L*. *pneumophila* have been acquired by inter-kingdom horizontal gene transfer from eukaryotic hosts, we sought to identify nutrient transporters in *L*. *pneumophila* based on their similarity to the human solute carrier (SLCs) transporters due to the lack of well annotated amoebal genomes^54,55^. This superfamily of transporters consists of over 65 families, grouped based on substrate specificity and tissue tropism^56^. They are considered to be part of a larger, evolutionarily conserved group of transporters known as the Major Facilitator Superfamily (MFS)^57,58^.

Eleven putative amino acid SLC-like transporters were identified, including a citrate transporter, 7 amino acids transporters and 2 glucose transporters. We focused our studies on the two putative SLC-like glucose transporters, LstA and LstB, to further understand the import of glucose and its role in intracellular replication of *L*. *pneumophila*.

## Results

### Identification of human SLC-like transporters in *L*. *pneumophila*

We utilized BLAST search of the *L*. *pneumophila* Philadelphia strain genome against all human SLC transporters of amino acids and glucose. In addition, since *L*. *pneumophila* utilizes pyruvate and citrate to feed its TCA cycle, we search for similarity of the *L*. *pneumophila* genome to the mono and tri-carboxylates SLC13 and 25 transporters^22,26^. Using the primary amino acid sequences from the human amino acid transporter families, SLC1, 3, 7, 17, 36, 38, and 43 against the genome of *L*. *pneumophila* strain AA100/130b, eight putative SLC-like amino acid transporters in *L*. *pneumophila* were identified by BLAST with similarity of 56–42% and identity of 25–37% (Table 1). In addition, one putative SLC-like transporter of tricarboxylates similar to the SLC13 family, *lpg2876* (24%/51%), was identified. In addition, using the primary amino acid sequence from the human SLC2 and SLC5 family, we identified two putative SLC-like glucose transporters, *lpg0421* (33%/50%) and *lpg1653* (30%/48%) (Table 1). Structural modeling of these proteins was done using the Iterative Threading Assembly Refinement (I-TASSER) server, which is a bioinformatics algorithm for predicting three-dimensional structure based on fold recognition^59–61^. Structural alignment was performed using TM-align, an algorithm that uses known or predicted protein structure to align proteins and determine structural similarity^59–61^. A TM-score is given for each alignment where, 1.0 indicates an exact copy, 0.0 −<0.3 indicates random structural similarity, and 0.5 −<1.0 indicating shared structural topology^61^. *L*. *pneumophila* proteins were compared to the human SLCs to determine structural homology, as measured by TM-scores (Table 1). Structural comparisons of these SLC-like transporters of *L*. *pneumophila* with human SLCs showed high structural similarity (TM-scores 0.78–0.977) (Table 1 and see Supplementary Fig. S1). Few homologs of mammalian SLCs have been identified within amoebae, which also can be used to identify *L*. *pneumophila* transporters; these are designated as CtrABC in *Dictyostelium discodium* (see Supplementary Fig. S2). We have designated these putative transporters as *Legionella* SLC-like transporters, LstA-K (Table 1).

**Table 1:** SLC-like putative proteins in *L*. *pneumophila* are homologous human SLCs.

	Amino acid identity (BLAST)	Amino acid similarity (BLAST)	Putative substrates	Representative SLC, TM-score
LstA (Lpg0421)	33%	50%	Glucose and other monosaccharides	SLC2a1 (0.903)
LstB (Lpg1653)	30%	48%	Glucose and other monosaccharides	SLC2a1 (0.922)
LstC (Lpg0026)	37%	56%	Cationic amino acids (arginine, lysine, ornithine)	SLC7a1 (0.953)
LstD (Lpg0049)	25%	44%	Cationic amino acids (arginine, lysine, ornithine)	SLC7a5 (0.954)
LstE (Lpg0228)	25%	42%	Cationic amino acids (arginine, lysine, ornithine)	SLC7a1 (0.848)
LstF (Lpg0281)	25%	53%	Cationic amino acids (arginine, lysine, ornithine)	SLC7a1 (0.985)
LstG (Lpg0970)	25%	44%	Cationic amino acids (arginine, lysine, ornithine)	SLC7a4 (0.855)
LstH (Lpg1691)	27%	45%	Cationic amino acids (arginine, lysine, ornithine)	SLC7a2 (0.756)
LstI (Lpg2245)	25%	53%	Alanine, serine, cysteine, and threonine	SLC1a4 (0.961)
LstJ (Lpg0886)	29%	54%	Neutral amino acids (glutamine, asparagine)	SLC1a5 (0.912)
LstK (Lpg2876)	24%	51%	Succinate, citrate, isocitrate, α-ketoglutarate	SLC13a3 (0.931)

Eleven transporters in *L*. *pneumophila* were identified by BLAST amino acid sequence homology with human SLC transporters. Predicated structures, generated by I-TASSER, were used to determine structural similarity with human SLCs by TM-score.

**Figure 1:**
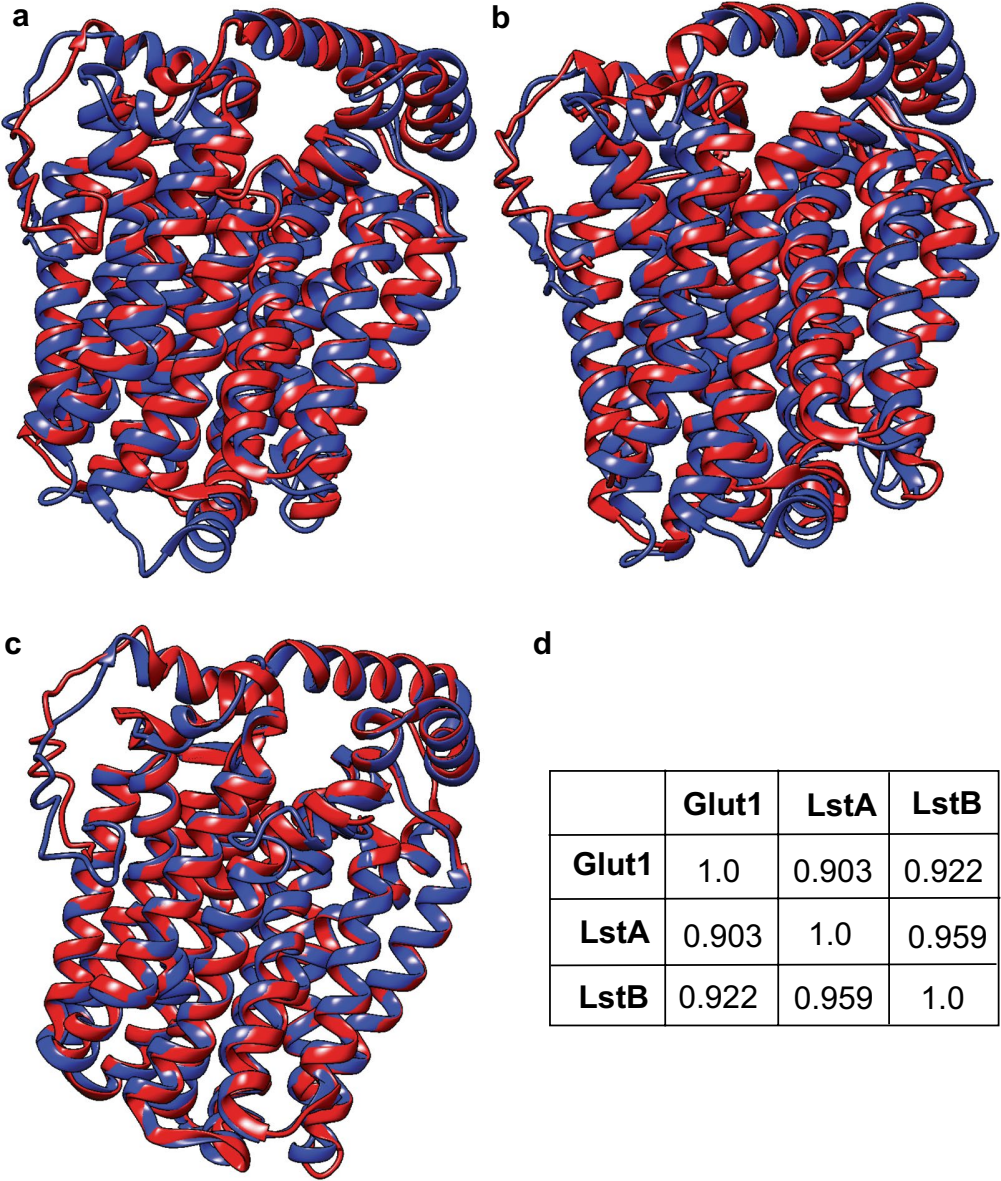
The predicted structure of two putative glucose transporters, LstA and LstB, of *L*. *pneumophila*. Structural alignment between (**a**) Glut1 (blue) and LstA (red) (**b**) Glut1 (blue) and LstB (red), and (**c**) LstA (red) and LstB (blue), are shown using TM-align. (**d**) TM-scores, indicating structural similiarity between Glut1, LstA, and LstB, as calculated by TM-align.

Since the uptake and the role of glucose in intracellular growth and metabolism of *L*. *pneumophila* is not well understood, we focused our studies on the two putative SLC-like transporters of *L*. *pneumophila* that shared strong structural similarity to SLC2a1/Glut1, LstA and LstB (Fig. 1a,b). LstA of *L*. *pneumophila* and Glut1 of humans have a TM-score of 0.903, and LstB and Glut1 a TM-score of 0.922, indicating very strong structural similarity (Fig. 1a,b,d). Members within the Glut family do not share this degree of similarity (Glut1 and Glut3, TM-score of 0.88). When comparing alignment of LstA to LstB, the TM-score is 0.959, indicating potential redundancy (Fig. 1c,d). These two *L*. *pneumophila* proteins are smaller in size than their human counterpart proteins but the secondary structural alignment is conserved. LstA and LstB are likely members of the major facilitator superfamily (MFS), which are important transporters that have been maintained, in all domains of life, with little deviation through evolutionary history^57^. LstA (Lpg0421) has been previously designated as YwtG, but based on its eukaryotic SLC-like structure and function (see below) and as one of a large family of eukaryotic SLC-like proteins in *L*. *pneumophila*, we have designated it as LstA^47^.

### Glucose import by LstA and LstB

**Figure 2:**
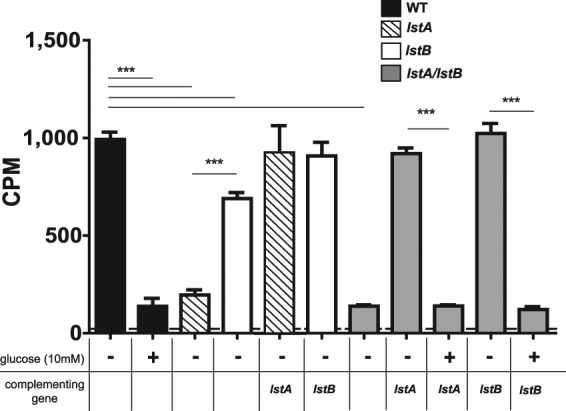
Glucose uptake in *L*. *pneumophila* by *lstA* and *lstB*. Uptake of ^14^C-glucose measured in counts per minute (CPM), of WT *L*. *pneumophila* (black), single mutants (white), and double mutants (grey), was determined. Addition of unlabeled glucose (10 mM) was used as a negative control (checkered). Data points represent mean CPM ± SD, n = 4 and are representative of three independent experiments.

Predicted substrate binding, by I-TASSER, for LstA and LstB indicate glucose as a putative substrate^59,60^. Given their high degree of structural similarity to Glut1, we hypothesized that both of these putative transporters were involved in the transport of glucose of *L*. *pneumophila*. To test if either LstA or LstB were required for the uptake of glucose, null mutants were generated and uptake of glucose was analyzed by liquid scintillation using ^14^C-glucose. *L*. *pneumophila* strains were grown to post-exponential phase in the presence of 0.1% uniformly labelled ^14^C-glucose. Broth grown WT *L*. pneumophila was able to effectively take up ^14^C-glucose *in vitro* (Fig. 2). As a control, excess, unlabeled glucose (10 mM) was added, which abolished uptake of ^14^C-glucose (Student *t-*test, *p* < 0.001) (Fig. 2). The *lstA* and *lstB* mutants had significantly reduced uptake of ^14^C-glucose compared to the WT strain (Student *t-*test, *p* < 0.001), but glucose uptake was more reduced in the *lstA* mutant (Fig. 2). Complementation of the single mutants with the respective gene on a plasmid (*lstA*.C and *lstB*.C) restored uptake of glucose to that of the WT strain levels (Fig. 2).

To determine whether LstA and LstB were redundant, a *lstA/lstB* double mutant, was generated. Loss of both transporters abolished uptake of ^14^C-glucose compared to the WT strain (Student *t-*test, *p* < 0.001) (Fig. 2). Upon supplementation of excess, unlabeled glucose (10 mM) uptake of labeled glucose was inhibited in the complemented double mutants (Student *t-*test, *p* < 0.001) (Fig. 2). Interestingly, complementation with a single transporter, (*lstA*.C or *lstB*.C) restored uptake of ^14^C-glucose to the double mutant similar to the WT strain levels (Fig. 2). These data show that LstA and LstB are glucose transporters.

### The LstA and LstB glucose transporters are required for growth in *Acanthamoeba polyphaga* and human monocyte-derived macrophages

**Figure 3:**
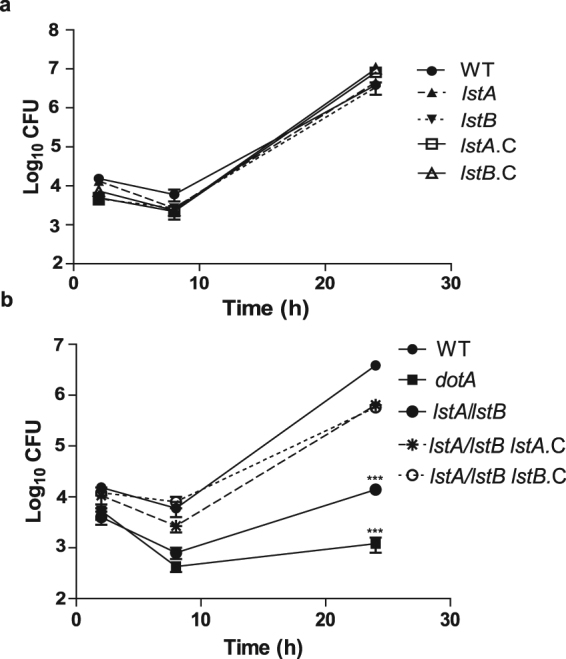
LstA and LstB are required for growth of *L*. *pneumophila* in amoebae. Intra-vacuolar replication of the (**a**) the WT strain; the two single transporter mutants, *lstA* and *lstB*; and the complemented single mutant, *lstA*.C and *lstB*.C, was determined in *A*. *polyphaga*. (**b**) The WT strain; the *dotA* mutant; the double mutant, *lstA/lstB*; and the complemented double mutant, *lstA/lstB lstA*.C and *lstA/lstB lstB*.C was also determined in *A*. *polyphaga*. The number of CFUs was determined at 2, 8, and 24 hrs post-infection. Data points represent mean CFUs ± SD, n = 3 and are representative of three independent experiments.

**Figure 4:**
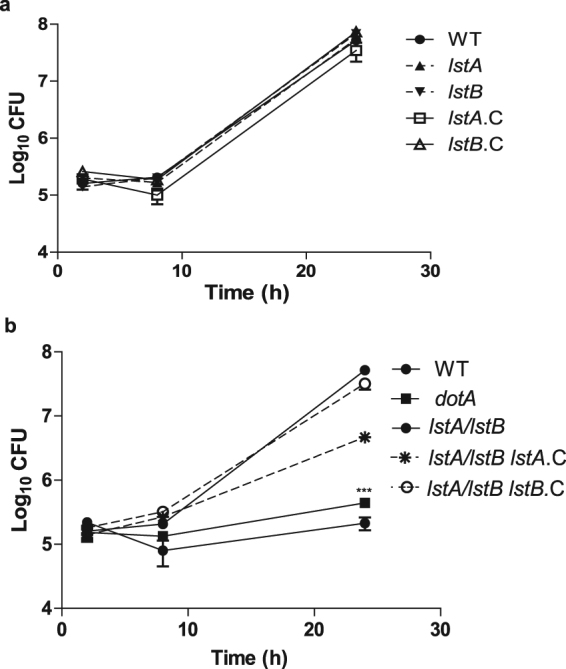
LstA and LstB are required for growth of *L*. *pneumophila* in hMDMs. Intra-vacuolar replication of the (**a**) the WT strain; the two single transporter mutants, *lstA* and *lstB*; and the complemented single mutant, *lstA*.C and *lstB*.C, was determined in hMDMs. (**b**) The WT strain; the *dotA* mutant; the double mutant, *lstA/lstB*; and the complemented double mutant, *lstA/lstB lstA*.C and *lstA/lstB lstB*.C was also determined hMDMs. The number of CFUs was determined at 2, 8, and 24 hrs post-infection. Data points represent mean CFUs ± SD, n = 3 and are representative of three independent experiments.

We determined intracellular replication of glucose transporter single mutants, *lstA* and *lstB*, and the double mutant *lstA/lstB* in human monocyte-derived macrophages (hMDMs) and *A*. *polyphaga*. Single transporter null mutants, *lstA* and *lstB*, replicated similarly to WT *L*. *pneumophila* in *A*. *polyphaga* or hMDMs (Figs 3a and 4a), which is consistent with the idea that they are redundant transporters. Given that the *lstA/lstB* double mutant resulted in a severely diminished uptake of glucose, we determined the ability of the double mutant to replicate intracellularly. *In vitro* cultures of *lstA/lstB* grew similarly to the WT strain (see Supplementary Fig. S3).

Interestingly the *lstA/lstB* double mutant was severely defective for growth in *A*. *polyphaga* and hMDMs (Two-way ANOVA, *p* < 0.001) (Figs 3b and 4b). Complementation of the double mutant, with individual single transporters, *lstA*.C or *lstB*.C, restored intracellular growth of the double mutant in *A*. *polyphaga* and hMDMs to almost that of WT *L*. *pneumophila* (Figs 3b and 4b). These data show that the two glucose transporters, LstA and LstB, are required for intracellular growth of *L*. *pneumophila* within hMDMs and *A*. *polyphaga* and these two transporters are most likely to be redundant in their function to import glucose^47^. Our data show that uptake of glucose is required for intracellular replication of *L*. *pneumophila* within evolutionarily distant host cells.

## Discussion

*L*. *pneumophila* generates copious amounts of host amino acids for carbon and energy but it is also reliant on host glucose^46,47,62^. Hauslein *et al*. described *L*. *pneumophila* metabolism as being “bipartite”, where amino acids serve as the major energy supply in the exponential phase and carbohydrates at the post-exponential phase are used in anabolic processes^48^. The role of glucose during intracellular infection can be difficult to study; methods for altering the levels of glucose affect the host cells, which may have detrimental effects on intracellular growth of *L*. *pneumophila* independent of the glucose level. The glucose analog, 2-deoxy-D-glucose (2-DG) causes autophagy in macrophages^63^. Increasing the levels of glucose in macrophages increases the inflammatory response, while starving cells of mimics glucose treatment with 2-DG^64,65^. Therefore, removing the *L*. *pneumophila*’s ability to access glucose by deletion of glucose transporters will best highlight the intracellular need for glucose without altering the host cell response to infection.

Our findings indicate that *L*. *pneumophila* utilizes two redundant glucose transporters, LstA and LstB, both of which transport glucose, and are required for growth within hMDMs and *A*. *polyphaga*. Surprisingly, the need for intracellular glucose is immediate, despite the fact that glucose is thought to be imported by *L*. *pneumophila* at the post-exponential phase and is thought to be primarily used in the late stages of growth for PHB synthesis^46,47,49,50^. Consistent with this idea, *lstA* is highly upregulated in the post-exponential growth phase *in vitro*, when glucose is being utilized^47,66^. However, *lstB* expression remains unchanged throughout the growth phases^66^. This could represent dual usages for glucose; LstB could transport low basal levels of glucose throughout intracellular growth while LstA transports large amounts of glucose when the demand has increased during late stages of growth^46,47^. Interestingly, LstA has been shown to be induced intracellularly during post-exponential growth of *L*. *pneumophila* in THP and *Acanthamoeba castellanii*, relative to growth *in vitro*^66,67^. This supports the idea that glucose is required for intracellular replication but not for *in vitro* growth of *L*. *pneumophila*^47^. However, loss of either of the two transporters, *lstA* or *lstB*, is not sufficient to affect intracellular growth, which is most likely due to functional redundancy.

LstA is situated adjacent to a glucose utilization gene cluster, which is important for the catabolism of glucose via the ED pathway^46,47^. Conflicting reports have shown that the gene cluster is required for growth in A549, A/J mouse macrophages, and *A*. *culbertsoni*, when mutated in *L*. *pneumophila* strain AM511^47^. However, in the Paris strain of *L*. *pneumophila*, deletion of one of the genes, *zwf*, does not result in a growth defect in *Acanthamoeba castellanii*^46^. Additionally, the genetic organization of the gene cluster in the AM511 and the Paris strains is different^46,47^. The *lstA* gene is 113 bp downstream of the 3′ end of *eda* of the glucose utilization gene cluster, which is sufficient for *lstA* to have its own promotor. The genetic organization of *lstA* is conserved among the Paris and AM511 strains, as well as our strain, AA100/130b.

The glucose transporter LstB/Lpg1653 is part of a myo-inositol catabolism gene cluster in *L*. *pneumophila*^68^. Although, LstB has been shown to transport inositol, inositol transporters are capable of transporting glucose, which is molecularly similar and acts as a competitive inhibitor of myo-inositol transport^68–70^. Therefore, it is possible that LstB has dual, or multi-, substrate specificity. Within *L*. *pneumophila*, myo-inositol is also metabolized into acetyl-CoA, which could also support the generation of PHB^68^.

Supplementation of glucose *in vitro* does not enhance the growth of *L*. *pneumophila*^47^. This may suggest the intracellular requirement for glucose does not support replication as a source of carbon and energy. Given that PHB is generated from glucose and is essential for survival outside of the host, lack of glucose uptake could be triggering stress response genes that prevent *L*. *pneumophila* from replicating^49^. If this were the case, starving host cells of glucose, would prevent the replication of intracellular *L*. *pneumophila*; however, starving host cells of glucose triggers cell death by autophagy^71^. Glucose is an important requirement for generating a reactive oxygen species (ROS) by amoebae and human macrophages in response to invading pathogens^64,65^. Uptake of glucose by *L*. *pneumophila* could serve dual purposes of sequestering glucose from the host, to dampen the immune response and to provide the precursor for PHB.

In summary, we have identified two redundant glucose transporters, LstA and LstB, which are required for intracellular replication of *L*. *pneumophila* in macrophages and amoebae. The requirement for glucose uptake by *L*. *pneumophila* is essential for intracellular growth in hMDMs and *A*. *polyphaga*, but not during growth *in vitro*. This presents an interesting question in *L*. *pneumophila* biology; why is glucose import required only during intracellular replication? This should be the focus of future studies.

## Materials and Methods

### Strains and cell lines

*L*. *pneumophila* strain AA100/130b (ATCC BAA-74) and the T4SS-deficient mutant, *dotA* were grown on Buffered Charcoal Yeast Extract (BCYE) agar, as we previously described^72^. To generate isogenic mutants in *lstA* and *lstB*, ~2 kb of flanking DNA on either side was amplified using primers listed in Table S2 and cloned into the shuttle vector, pBCSK + lstAKO and pBCSK + lstBKO (Table S1). The entire gene of either *lstA* or *lstB* was deleted via inverse PCR using the primers listed in Table S2, resulting in pBCSK + lstAKOi and pBCSK + lstBKOi (Table S1). The kanamycin cassette from the Ez-Tn5 transposon was amplified using primers listed in Table S2 and the resulting PCR product was subcloned into in pBCSK + lstAKOi and pBCSK + lstBKOi between the flanking regions of either *lstA* or *lstB*, using standard molecular procedures, resulting in pBCSK + lstAKAN and pBCSK + lstBKAN (Table S1). Each resulting plasmid was independently introduced into *L*. *pneumophila* AA100/130b via natural transformation, as we previously described^73^. After three days, natural transformants were recovered by plated on BCYE supplemented with 50 μg/ml kanamycin, to generate *L*. *pneumophila lstA* and *L*. *pneumophila lstB* (Table S2). To confirm deletion of either *lstB* or *lstB*, the forward primer for sequencing and the reverse primer for generation of the knockout, listed in Table S2 were used. To generate double mutants, a gentamycin cassette was amplified using primers listed in Table S2 and the resulting PCR product was subcloned into in pBCSK + lstAKOi between the flanking regions of either *lstA*, using standard molecular procedures, resulting in in pBCSK + lstAGENT (Table S1). The resulting plasmid was introduced into *L*. *pneumophila lstB* via natural transformation, as previously described^73^. After three days, natural transformants were recovered by plated on BCYE supplemented with 20 μg/ml kanamycin and 5 μg/ml gentamycin, to generate *L*. *pneumophila lstB/lstA* (Table S1). Deletions were confirmed using the same primers as described above.

To generate complement mutants of single deletions and double deletions, *lstA* or *lstB* with flanking upstream and downstream sequences were amplified by PCR using the primers listed in Table S2, and subcloned into pBCSK+, generating pBCSK + *lstA*.C and pBCSK-*lstB*.C (Table S1). The pBCSK + *lstA*.C plasmid was introduced into the *lstA* and *lstA/lstB* mutants and the pBCSK + *lstB*.C plasmid was introduced into the *lstB* and *lstA/lstB* mutants via electroporation as previously described (Table S1)^74^. All complement mutants were selected on BCYE plates supplemented with 5 μg/ml chloramphenicol, resulting in the following complement strains: *lstA*.C, *lstB*.C, *lstA/lstB lstA*.C, and *lstA/lstB lstB*.C (Table S1).

Human monocyte-derived macrophages (hMDMs) were isolated from healthy adult donors and cultured in RPMI 1640 (Corning) supplemented with 10% fetal bovine serum, as previously described^72^. All methods were carried out and approved in accordance to the University of Louisville Institutional Review Board guidelines and blood donors gave informed consent as required by the University of Louisville Institutional Review Board (IRB # 04.0358). U937 cells were cultured in RPMI 1640 (Corning) supplemented with 10% fetal bovine serum and *A*. *polyphaga* was cultured in PYG media, experiments were performed in PY media, as we previously described^72^.

### Structural comparison of glucose transporters

Predicted structures were generated via I-TASSER server from the Zhang Lab (https://zhanglab.ccmb.med.umich.edu/I-TASSER/)^59,60,75^. Structures generated from I-TASSER were aligned using TM-align to determine structural alignment and TM-scores for similarity (https://zhanglab.ccmb.med.umich.edu/TM-align/)^61,76^.

### Glucose uptake assay

Uptake of glucose was assayed by growing WT *L*. *pneumophila*, *lstA*, *lstB*, and complemented mutant strains individually in the presence of ^14^C-label glucose (specific activity, 3.3 MBq/μmol) (PerkinElmer) and the presence of glucose followed into the acid-insoluble fraction, as previously described^47^. One milliliter cultures were grown in Buffered Yeast Extract (BYE) broth supplemented with 0.1% D-[U-^14^C] glucose (specific activity, 3.3 MBq/μmol) at 37 °C, shaking to post-exponential phase (>OD_550_ 2.0). For control, 10 mM sterile glucose was added to broth cultures with 0.1% D-[U-^14^C] glucose. Samples normalized to 10^9^ bacteria in 5 ml 1% Triton X-100 for 30 mins, and then incubated for 30 mins with 5 ml of chilled 10% (w/v) trichloroacetic acid, on ice. To capture radioactivity, samples were filtered through nitrocellulose filters (0.45-μm pore size; Milipore) and rinsed three times with chilled 5% trichloroacetic acid. Radioactivity of the whole sample was determined by liquid scintillation (Tri-Carb 2910 TR, PerkinElimer) with BetaBlend scintillation cocktail (MP Biochemical).

### Intracellular replication

*L*. *pneumophila*; the isogenic single mutants, *dotA*, *lstA* and *lstB*; the double isogenic mutant *lstB/lstA*; and the complement mutants *lstA*.C, *lstB*.C, *lstB/lstA lstB*.C, and *lstB/lstA lstA*.C were grown to post-exponential phase on BCYE plates at 37 °C prior to infection and used to infect hMDMs or *A*. *polyphaga*, as previously described^72,77^. A total of 1 × 10^5^ host cells per well were plated into 96 well plates and infected with *L*. *pneumophila* at an MOI of 10 for 1 h then treated with gentamycin to kill remaining extracellular bacteria, as previously described^72,77^. Host cells were lysed with sterile water (hMDMs) or 0.02% Triton X-100 (*A*. *polyphaga*) at various timepoints over a 24 h timecourse and *L*. *pneumophila* CFUs were determined by plating serial dilutions onto BCYE agar.

### Data availability

All data generated or analysed during this study are included in this published article (and its Supplementary Information files).

## Electronic supplementary material

Supplementary material
