# Supplementary material for: Mammalian Solute Carrier (SLC)-like transporters of Legionella pneumophila
Source: Sci Rep. 2018 May 29;8:8352. doi: 10.1038/s41598-018-26782-x (PMC5974234; doi:10.1038/s41598-018-26782-x)
Supplement: Supplementary file 1 — Supplementary material [file 41598_2018_26782_MOESM1_ESM.docx]

**Supplementary material for:**

**Mammalian Solute Carrier (SLC)-like transporters of *Legionella pneumophila***

Ashley Best^1^, Snake Jones^1^, and Yousef Abu Kwaik*^1,2^

^1^Department of Microbiology and Immunology, College of Medicine, ^2^Center for Predictive Medicine, University of Louisville, Louisville, KY

*To whom correspondence should be addressed: [abukwaik@louisville.edu](mailto:abukwaik@louisville.edu)


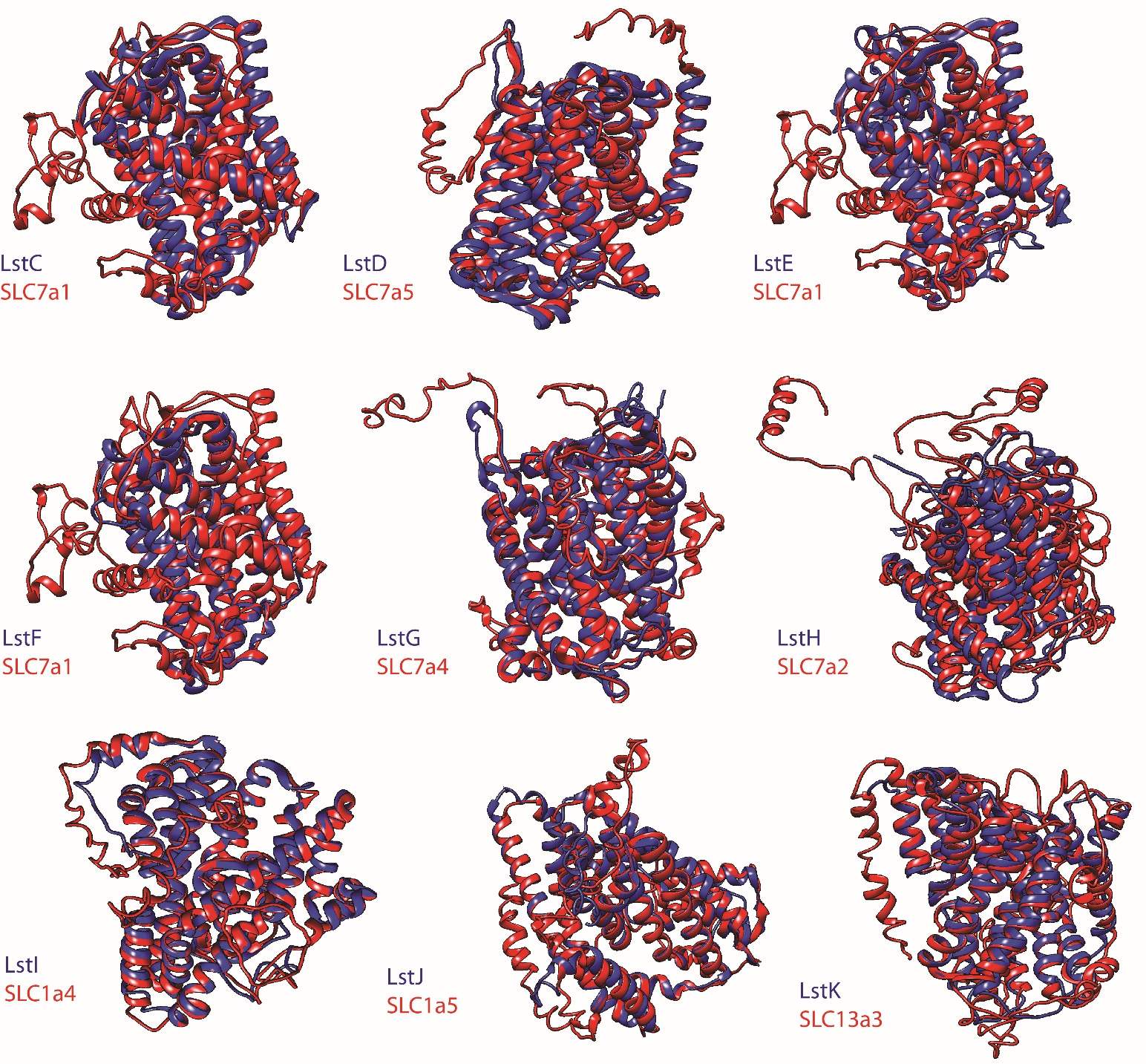


**Figure S1. Structural alignment of human SLCs and SLC-like proteins in *L. pneumophila*.** I-TASSER predicted structures of SLC-like proteins aligned, via TM-align, with human SLCs based on highest TM-score, indicate strong structural similarity.


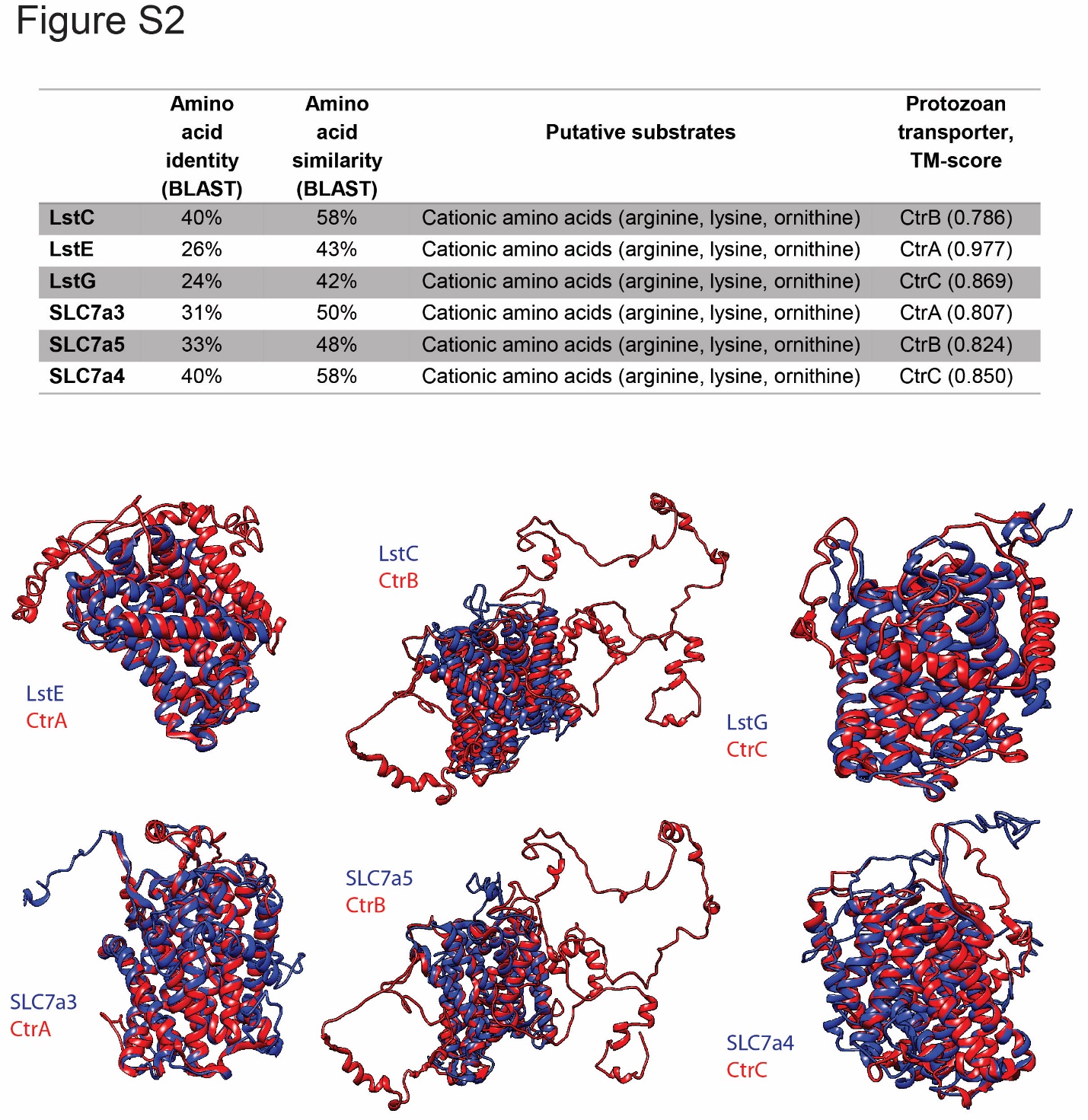


**Figure S2. Similarity and identity of *L. pneumophila* SLC-like transporters and protozoan SLC-like transporters.** SLC-like amino acid transporters are present in *Dictyostelium discodium*. These protozoan transporters share structural homology with the human SLC transporters and with *L. pneumophila* SLC-like transporters. A) Degrees of amino acid sequence identity and similarity between these transporters are shown in and B) structural alignment.

**
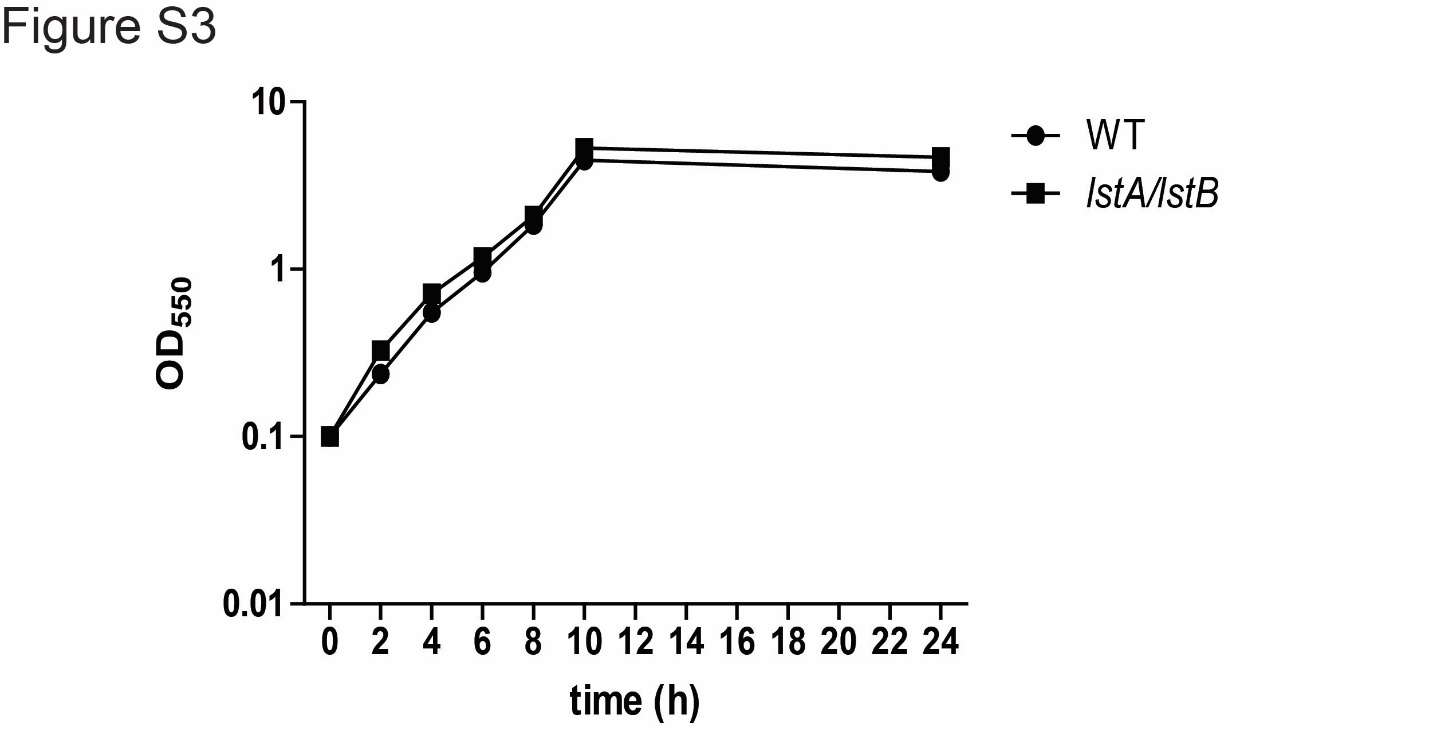
**

**Figure S3. The *lstA* and *lstB* mutants grow similar to WT *in vitro*.** Overnight cultures of WT and *lstA/lstB* in BYE broth diluted to OD_550nm_ of 0.05 and grown at 37°C for 24 hrs. Growth rates were determined by measuring OD_550nm_ every 2 hrs, for 12 hrs, then again at 24 hrs post-inoculation. Data are representative of three independent experiments.

**Table S1. Bacterial strains and plasmids used in this study**

| Strain or plasmid | Genotype |
| --- | --- |
| *L. pneumophila* |  |
| AA100/130b |  |
| *dotA* |  |
| *lstA* | *lstA*, Kan^r^ substitution |
| *lstB* | *lstB*, Kan^r^ substitution |
| *lstA/lstB* | *lstB*, Kan^r^ substitution *lstA*, Gent^r^ substitution |
| *lstA.*C | *lstA* complemented with pBCsk+*lstA*.C |
| *lstB*.C | *lstB* complemented with pBCsk+*lstB*.C |
| *lstB/lstA lstA.C* | *lstB/lstA* complemented with pBCsk+*lstA*.C |
| *lstB/lstA lstB.C* | *lstB*/*lstA* complemented with pBCsk+*lstB*.C |
|  |  |
|  |  |
| Plasmids |  |
| pBCsk- |  |
| pBCsk+*lstA*KO | 2kB region upstream and downstream of *lstA* |
| pBCsk+*lstB*KO | 2kB region upstream and downstream of *lstB* |
| pBCsk+*lstA*KOi | pBCSK+*lstA*KO with *lstA* removed |
| pBCsk+*lstB*KOi | pBCKSK+*lstB*KO with *lstB* removed |
| pBCsk+*lstA*Kan^r^ | pBCSK+*lstA*KO with *lstA* replaced with kanamycin resistance cassette |
| pBCsk+*lstB*Kan^r^ | pBCSK+*lstB*KO with *lstA* replaced with kanamycin resistance cassette |
| pBCsk+*lstA*Gent^r^ | pBCSK+*lstA*KO with *lstA* replaced with gentamycin resistance cassette |
| pBCsk+*lstA*.C | *lstA*, with 100bp promotor region |
| pBCsk+*lstB*.C | *lpg1649-lstB*, with 100bp promotor region |
|  |  |

**Table S2. Primers used in this study**

| Primer | Sequence |
| --- | --- |
| lstA-KO F | GGTACCATATGATTTTGATCGATTGT |
| lstA-KO R | GAGCTCAAGGAAGCACATAAATTAAAA |
| lstB-KOF | GGTACCAAAACATTCAATACTTCAACA |
| lstB-KO R | GAGCTCTCAGTGAATATAAATAAACGCT |
| lstA inverse F | TATAAATTTTCCTATGATGCCC |
| lstA inverse R | TTTTCACTCCTTGATTTTAAATTCT |
| lstB inverse F | GTAACTATCTGTCCCTAATGAAAATTC |
| lstB inverse R | TCATGAAAAAGAAAATATGTCGAAT |
| Kan F, R | /5Phos/CTGTCTCTTATACACATCTCAA |
| lstA sequence primer | TATCAAGAATCATAACTGGTCTTT |
| lstB sequence primer | TAATTTTTCTGCCCAATTCCTATCGA |
| Gent F | /5Phos/ATGTTACGCAGCAGCAACGAT |
| Gent R | /5Phos/TTAGGTGGCGGTACTTGGGTC |
| lstA-C F | GGTACCTTCGGGTTGAAAAAGCGT |
| lstA-C R | GAGCTCCGTGTCAGTCAAAGATAACA |
| lstB-C F | GGTACCGGATAATCTCCTTATTATATTG |
| lstB-C R | AGCTCATGCTTTGGTCAATTAAC |
